# Paraventricular hypothalamic vasopressin neurons induce self-grooming in mice

**DOI:** 10.1186/s13041-022-00932-9

**Published:** 2022-05-23

**Authors:** Md Tarikul Islam, Takashi Maejima, Ayako Matsui, Michihiro Mieda

**Affiliations:** Department of Integrative Neurophysiology, Graduate School of Medical Sciences, Kanazawa University, 13-1 Takara-machi, Kanazawa, Ishikawa 920-8640 Japan

**Keywords:** Self-grooming, Vasopressin, Paraventricular hypothalamic nucleus, Channelrhodopsin, DREADDs, Repetitive behavior

## Abstract

Self-grooming plays an essential role in hygiene maintenance, thermoregulation, and stress response. However, the neural populations involved in self-grooming remain largely unknown. The paraventricular hypothalamic nucleus (PVH) has been implicated in the regulation of self-grooming. Arginine vasopressin-producing neurons are among the major neuronal populations in the PVH (PVH^AVP^), which play important roles in water homeostasis, blood pressure regulation, feeding, and stress response. Here, we report the critical role of PVH^AVP^ neurons in the induction of self-grooming. Optogenetic activation of PVH^AVP^ neurons immediately induced self-grooming in freely moving mice. Chemogenetic activation of these neurons also increased time spent self-grooming. In contrast, their chemogenetic inhibition significantly reduced naturally occurring self-grooming, suggesting that PVH^AVP^-induced grooming has physiological relevance. Notably, optogenetic activation of PVH^AVP^ neurons triggered self-grooming over other adaptive behaviors, such as voracious feeding induced by fasting and social interaction with female mice. Thus, our study proposes the novel role of PVH^AVP^ neurons in regulating self-grooming behavior and, consequently, hygiene maintenance and stress response. Furthermore, uncontrolled activation of these neurons may be potentially relevant to diseases characterized by compulsive behaviors and impaired social interaction, such as autism, obsessive–compulsive disorder, and anorexia nervosa.

**Supplementary Information:**

The online version contains supplementary material available at 10.1186/s13041-022-00932-9.

## Introduction

Animals perform maintenance behaviors for their basic subsistence. Such behaviors include drinking, feeding, washing, grooming, preening, and sleeping. In rodents, self-grooming is a vital maintenance behavior characterized by scratching, licking, or biting the fur, body, whiskers, feet, or genitals [1]. Rodents groom themselves to keep the body clean, maintain body temperature, protect the body from foreign materials, and reduce stress levels [2]. They feed, drink, walk, and explore during the remaining awake time.

In addition to serving the functions of hygiene maintenance and thermoregulation, self-grooming has an essential role in stress response [3]. Self-grooming acts as an adaptive behavior to avoid over-response to stress [1, 4, 5]. On the other hand, over-grooming in rodents is a repetitive, compulsive behavior comparable to obsessive thought or obsessive behavioral change characteristic to some psychiatric disorders, such as obsessive–compulsive disorder, obsessive eating disorder, and autism spectrum disorder [6–10]. Thus, unraveling the brain regions and neuronal populations regulating self-grooming is valuable for understanding the neurobiological basis of hygiene maintenance, stress management, and those psychiatric disorders.

In recent years, the limbic and hypothalamic neural circuits involved in self-grooming behavior have begun to emerge [5, 11–14]. The paraventricular hypothalamus (PVH) is one of the regions of interest. It is an autonomic control system well-known for its essential roles in metabolism, stress response, and body-fluid homeostasis through its projections to the hypophyseal endocrine system, the autonomic nervous system, and many other brain regions [15–17]. Local electrical or pharmacological activation of PVH and surrounding regions have been reported to initiate self-grooming [18]. PVH contains multiple types of neurons that have different physiological functions. Among them, corticotropin-releasing hormone (CRH)-producing neurons (PVH^CRH^) form the central axis of stress response and have been demonstrated to increase self-grooming in mice upon their optogenetic activation [13]. Consistently, central administration of CRH or adrenocorticotropic hormone (ACTH) elicits self-grooming [19, 20].

AVP-producing neurons are another major neuronal population in the PVH. PVH^AVP^ neurons play a role in water homeostasis, blood pressure regulation, food intake regulation, social interactions, and stress response [21–27]. In addition, intracerebroventricular administration (ICV) of AVP has been demonstrated to increase grooming [27–30]. However, the involvement of PVH^AVP^ neurons in the regulation of grooming behavior remains unknown. In this study, we used optogenetics and chemogenetics to address whether these neurons affect self-grooming.

## Results

### Optogenetic activation of PVH^AVP^ neurons induces self-grooming in freely moving mice

**Fig. 1:**
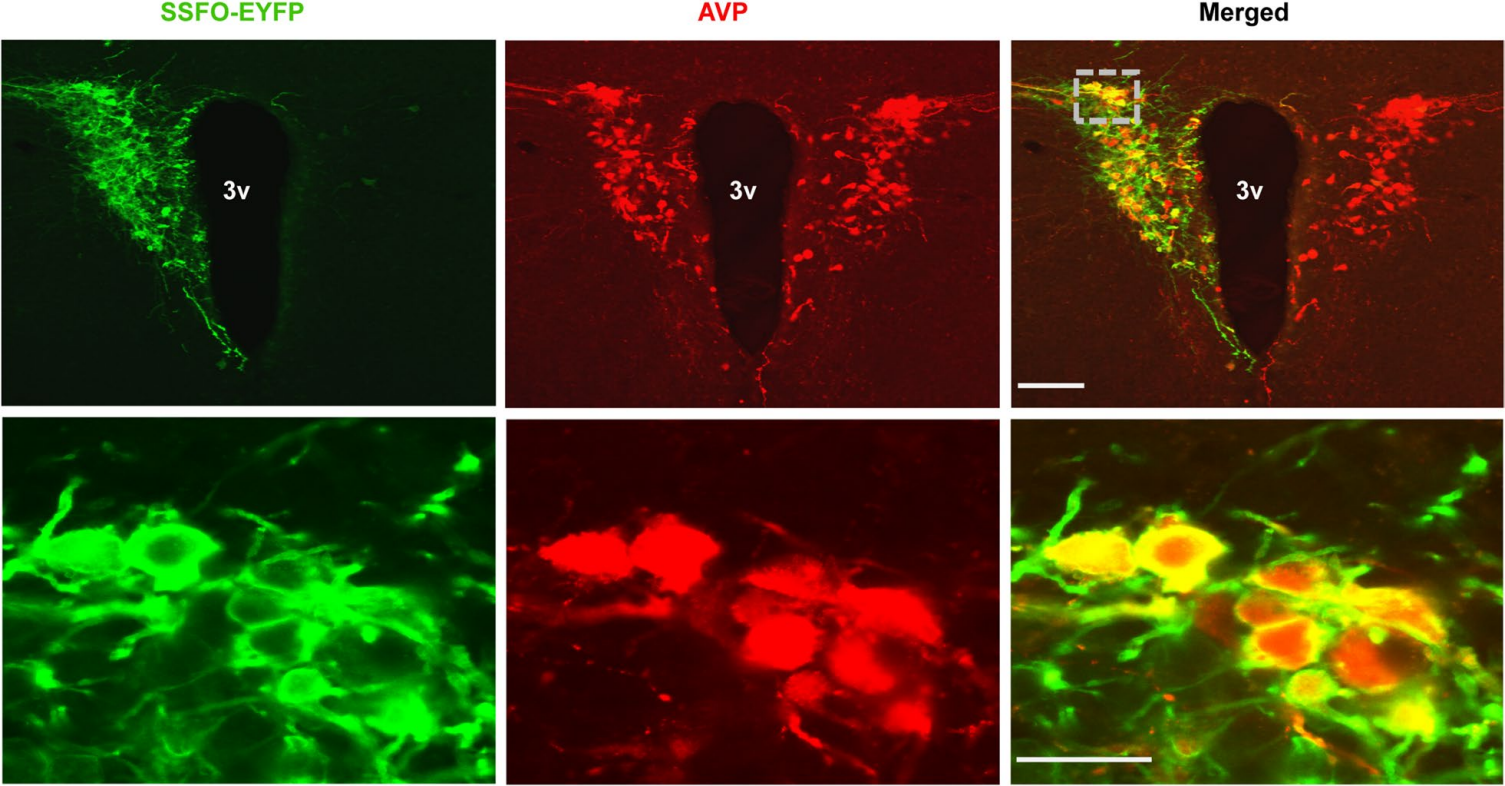
SSFO is expressed specifically in PVH^AVP^ neurons. A representative coronal brain section containing the PVH prepared from an *Avp-Cre* mouse with a focal injection of AAV-*EF1α-DIO-SSFO-EYFP* in the PVH and double-stained with anti-AVP (red) and anti-GFP (green) antibodies. 3v, third ventricle. (Scale bar: 200 μm for upper panels, 50 μm for lower panels)

**Fig. 2:**
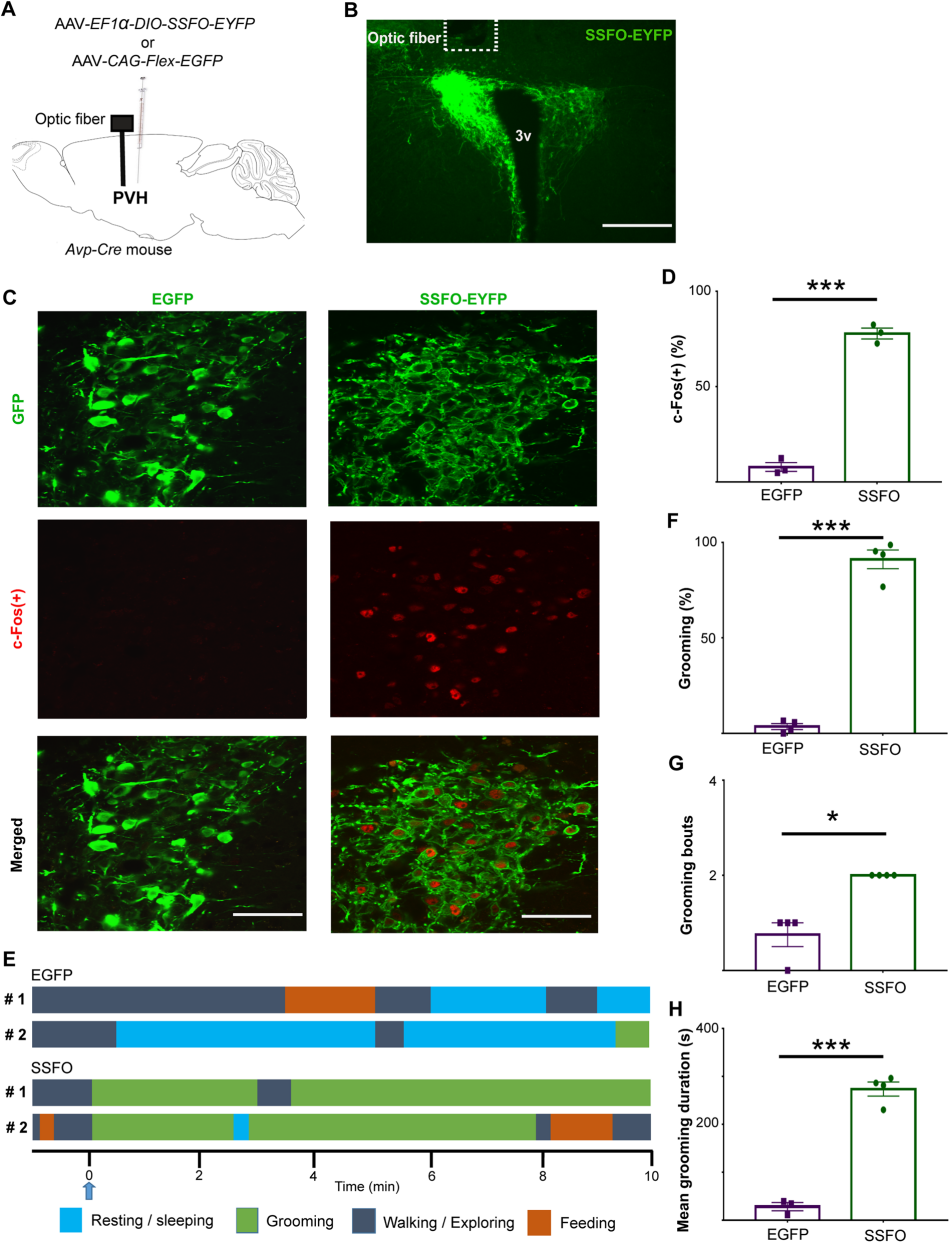
Optogenetic activation of PVH^AVP^ neurons increases self-grooming in freely moving mice. **A** Schematic representation of viral vector injection strategy and optic fiber placement above the PVH in *Avp-Cre* mice. **B** A representative coronal brain section of the PVH prepared from an *Avp-Cre* mouse with a targeted injection of AAV-*EF1α-DIO-SSFO-EYFP* in the PVH. The position of an optic fiber implant is indicated by a dotted white line. 3v, third ventricle; Scale bar: 300 μm. **C** Representative coronal brain sections prepared from *Avp-Cre* mice expressing SSFO-EYFP or EGFP in the PVH 90 min after a blue-light stimulation (2 s). Slices were double-stained with anti-GFP (green) and anti-c-Fos (red) antibodies. Scale bar, 60 μm. **D** c-Fos expression was increased by SSFO-EYFP stimulation in PVH^AVP^ neurons (n = 3). **E** Time courses of behaviors of 2 mice expressing SSFO-EYFP and 2 mice expressing EGFP in PVH^AVP^ neurons subjected to blue-light illumination. The stimulation point is shown by a blue arrow and denoted as 0 min in the time course. **F** Time spent self-grooming, **G** number of grooming bouts, and **H** mean duration of grooming bouts for 10 min following blue-light illumination. Values are mean ± SEM; n = 4; *p < 0.05, ***p < 0.001 by Welch's t-test (**D**, **F**, **G** and **H**)

To test whether PVH^AVP^ neurons play a role in self-grooming, we took an optogenetic approach to activate these neurons. We used the stable step-function opsin (SSFO), a variant of channelrhodopsin 2 (ChR2), that remains active for 20–30 min once activated by blue light, mimicking the depolarized state upon enhanced excitatory inputs [31, 32]. To express SSFO specifically in PVH^AVP^ neurons, we unilaterally injected a Cre-On adeno-associated virus vector AAV-*EF1α-DIO-SSFO-EYFP* in the PVH of mice expressing Cre recombinase specifically in AVP neurons (*Avp-Cre* mice) [33] (Fig. 1). We first verified whether SSFO stimulation increases the firing rate of PVH^AVP^ neurons. Slice electrophysiology revealed that activation of SSFO in PVH^AVP^ neurons increased their firing rate (baseline: 4.5 ± 0.6 Hz vs. optogenetic activation: 9.8 ± 0.4 Hz) (Additional file 1: Fig. S1). We also examined in vivo the effects of SSFO stimulation on the activity of PVH^AVP^ neurons. A brief blue-light illumination to PVH^AVP^ neurons via an optic fiber significantly increased c-Fos expression in SSFO-EYFP-expressing PVH^AVP^ neurons compared to control EGFP-expressing PVH^AVP^ neurons (EGFP: 7.8 ± 2.3% vs. SSFO: 77.6 ± 2.9%) (Fig. 2A–D). Thus, PVH^AVP^ neurons can be activated optogenetically in vivo.

Then, we observed mouse behavior after an optogenetic activation of PVH^AVP^ neurons by videorecording in the light period. Upon optogenetic stimulation, freely moving mice immediately exhibited self-grooming (latency: 3.7 ± 0.4 s) (Additional file 2: Video S2). Furthermore, they drastically increased grooming behavior and spent most time grooming during 10 min of observation after stimulation (EGFP: 3.5 ± 1.5% vs. SSFO: 91.1 ± 4.9%) (Fig. 2E–H, Additional file 2: Video S2 and Additional file 3: Video S3). Thus, our result suggested that activation of PVH^AVP^ neurons induces self-grooming in mice.

Self-grooming is not a unitary behavior and contains multiple phases in which animals groom different body parts [1, 5, 34]. Furthermore, previous studies suggested that the patterns of self-grooming are variable and differentially associated with physical and emotional stress [5]. Thus, we compared the pattern of self-grooming induced by the optogenetic activation of PVH^AVP^ neurons with that of spontaneous ones. To do so, we dissected self-grooming behavior into four phases. Namely, (1) paw licking, (2) face/head grooming, (3) body grooming, and (4) leg/tail/genital grooming. During PVH^AVP^ neuron-induced self-grooming, mice spent paw licking for significantly longer at the expense of body/leg/tail/genital grooming than spontaneous self-grooming (Additional file 1: Fig. S2). Such a pattern of PVH^AVP^ neuron-induced self-grooming may resemble those of grooming caused by emotional stress in rats [5].

Previous studies have reported that some PVH^CRH^ neurons co-express AVP in rats, primarily upon adrenalectomy [35]. Therefore, we confirmed that PVH^AVP^ neurons stimulated optogenetically constituted a population distinct from PVH^CRH^ neurons in our experimental conditions. To do this, we injected a reporter AAV-*CAG-FLEX-EGFP* in the PVH of *Avp-Cre* mice. We immunostained brain sections prepared from these mice pretreated with colchicine, which was required to delineate cell bodies with an anti-CRH antibody. Only 3.8 ± 1.2% of EGFP-positive cells were also CRH-positive, whereas AVP-positive cells accounted for 90.6 ± 2.0% of EGFP-positive cells in the PVH (Additional file 1: Fig. S3). This result suggested that optogenetic induction of self-grooming we observed was caused by activation of PVH^AVP^ neurons but not by a part of PVH^CRH^ neurons.

### Chemogenetic activation of PVH^AVP^ neurons promotes self-grooming in freely moving mice

**Fig. 3:**
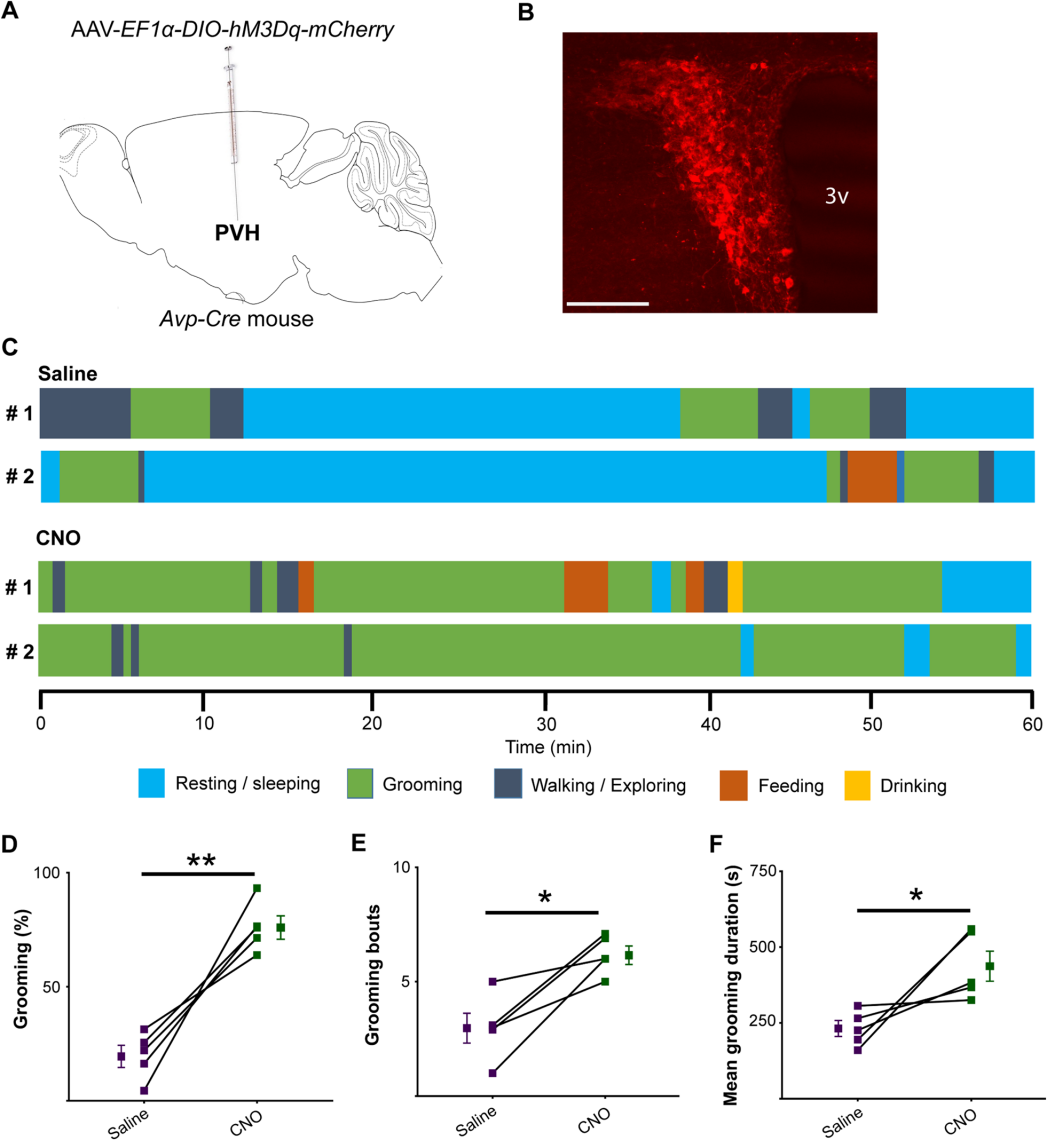
Chemogenetic activation of PVH^AVP^ neurons increases self-grooming in freely moving mice. **A** Schematic representation of viral vector AAV-*EF1α-DIO-hM3Dq-mCherry* injection in the PVH of an *Avp-Cre* mice. **B** A representative coronal brain section containing the PVH prepared from *Avp-Cre* mouse with a focal injection of AAV-*EF1α-DIO-hM3Dq-mCherry* in the PVH. 3v, third ventricle; Scale bar, 200 μm. **C** Time courses of behaviors of 2 mice expressing hM3Dq in PVH^AVP^ neurons following saline or CNO administration. **D** Time spent in self-grooming, **E** number of grooming bouts, and **F** mean duration of grooming bouts for 60 min following saline or CNO administration. Values are mean ± SEM; n = 5; *p < 0.05, **p < 0.005 by paired t-test

Next, we verified the finding of our optogenetic study by the chemogenetic approach. For chemogenetic activation, we expressed hM3Dq, an excitatory Designer Receptors Exclusively Activated by Designer Drugs (DREADD) [36], in PVH^AVP^ neurons by unilaterally injecting AAV-*EF1α-DIO-hM3Dq-mCherry* in the PVH of *Avp-Cre* mice (Fig. 3A and B). The excitatory effect of hM3Dq stimulation on these neurons was verified by slice electrophysiology, showing an increase of firing rate from 5.5 ± 0.9 Hz to 14 ± 1.8 Hz upon CNO application (Additional file 1: Fig. S4A and B). Chemogenetic activation of PVH^AVP^ neurons in vivo by CNO administration significantly increased time spent self-grooming compared to saline administration during 1 h of observation after administration from zeitgeber time (ZT) 3 to ZT4 (Saline: 19.9 ± 4.6% vs. CNO: 76.2 ± 4.8%) (Fig. 3C–F). Thus, our chemogenetic study further confirmed the ability of PVH^AVP^ neurons to promote grooming.

### Chemogenetic inhibition of PVH^AVP^ neurons reduces self-grooming in freely moving mice

**Fig. 4:**
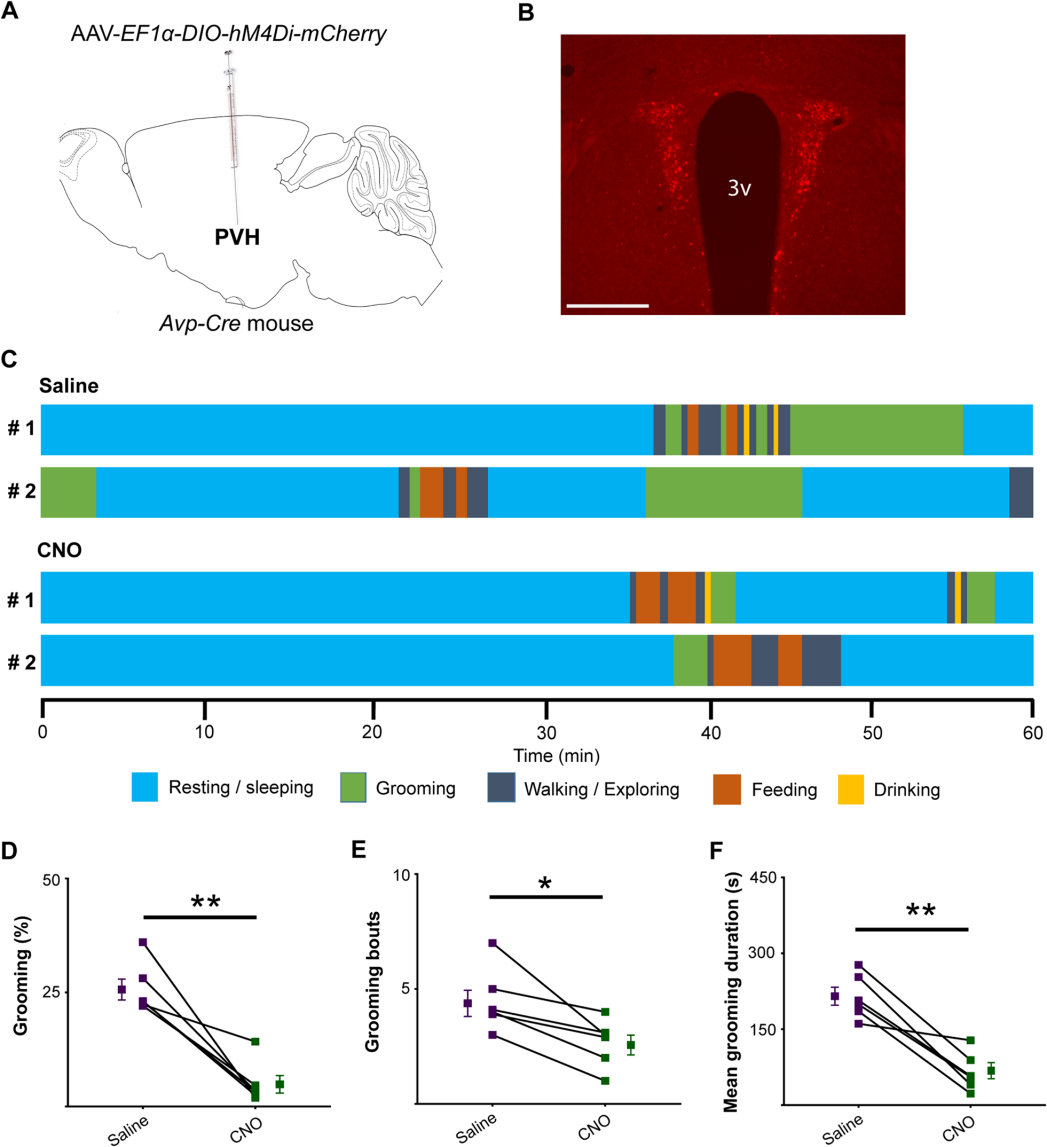
Chemogenetic inhibition of PVH^AVP^ neurons reduces self-grooming in freely moving mice. **A** Schematic representation of viral vector AAV-*EF1α-DIO-hM4Di-mCherry* injection in the PVH of *Avp-Cre* mice. **B** A representative coronal brain section containing the PVH prepared from an *Avp-Cre* mouse with bilateral focal injections of AAV-*EF1α-DIO-hM4Di-mCherry* in the PVH. 3v, third ventricle; Scale bar, 300 μm. **C** Time courses of behaviors of 2 mice expressing hM4Di in PVH^AVP^ neurons following saline or CNO administration. **D** Time spent in self-grooming, **E** number of grooming bouts, and **F** mean duration of grooming bouts for 60 min following saline or CNO administration. Values are mean ± SEM; n = 6; *p < 0.05, **p < 0.005 by paired t-test

We next examined whether inhibition of PVH^AVP^ neurons reduces naturally occurring self-grooming in the homecage. To do this, we expressed an inhibitory DREADD, hM4Di [36], in PVH^AVP^ neurons by bilaterally injecting AAV-*EF1α-DIO-hM4Di-mCherry* in the PVH of *Avp-Cre* mice (Fig. 4A and B). The inhibitory effect of hM4Di on these neurons was verified by slice electrophysiology, showing a decrease of firing rate from 7.6 ± 2.3 to 1.8 ± 1.3 Hz upon CNO application (Additional file 1: Fig. S4C and D). Chemogenetic suppression of PVH^AVP^ neurons by CNO administration significantly reduced self-grooming during 1 h of observation after administration from ZT11 to ZT12, when mice generally show an anticipatory increase of wakefulness before the onset of the dark period (Saline: 25.8 ± 2.2% vs. CNO: 4.9 ± 1.8%) (Fig. 4C–F). This result suggested that PVH^AVP^ neurons are involved in the physiological regulation of self-grooming.

### Optogenetic activation of PVH^AVP^ neurons causes self-grooming over voracious feeding induced by fasting

**Fig. 5:**
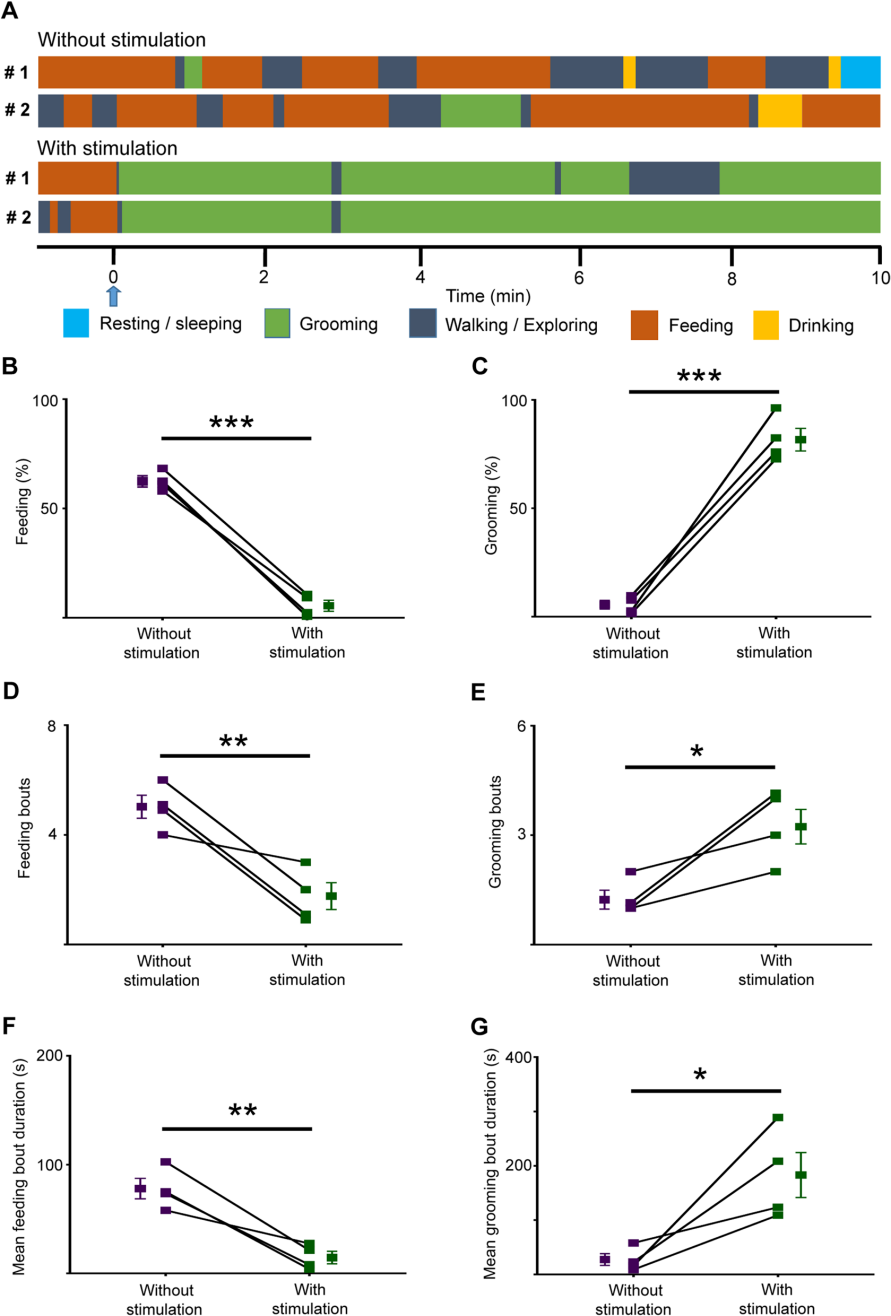
Optogenetic activation of PVH^AVP^ neurons causes self-grooming over voracious feeding induced by fasting. **A** Time courses of behaviors of 2 mice expressing SSFO-EYFP in PVH^AVP^ neurons during refeeding after ~ 24 h fasting without or with blue-light illumination (2 s). The stimulation point is shown by a blue arrow and denoted as 0 min in the time course. **B**–**G** Time spent in feeding (**B**) or grooming (**C**), number of feeding (**D**) or grooming bouts (**E**), mean duration of feeding (**F**) or grooming bouts (**G**) for 10 min of refeeding after ~ 24 h food deprivation. Values are mean ± SEM; n = 4; *p < 0.05, **p < 0.005, ***p < 0.001 by Welch's t-test

We next tested whether the optogenetic activation of PVH^AVP^ neurons could switch other adaptive behaviors triggered by physiological needs into self-grooming. We first examined fasting-induced feeding. Mice expressing SSFO-EYFP in PVH^AVP^ neurons were fasted for 24 h and then refed. Fasted mice spent most time feeding when refed (Fig. 5A, Additional file 4: Video S4). However, optogenetic activation of PVH^AVP^ neurons efficiently suppressed feeding and instead induced self-grooming (latency: 4.7 ± 1.2 s) (Fig. 5A–G, Additional file 5: Video S5). This result suggested that activation of PVH^AVP^ neurons prioritized self-grooming over hunger-induced feeding.

### Optogenetic activation of PVH^AVP^ neurons causes self-grooming over social interaction between male and female mice

**Fig. 6:**
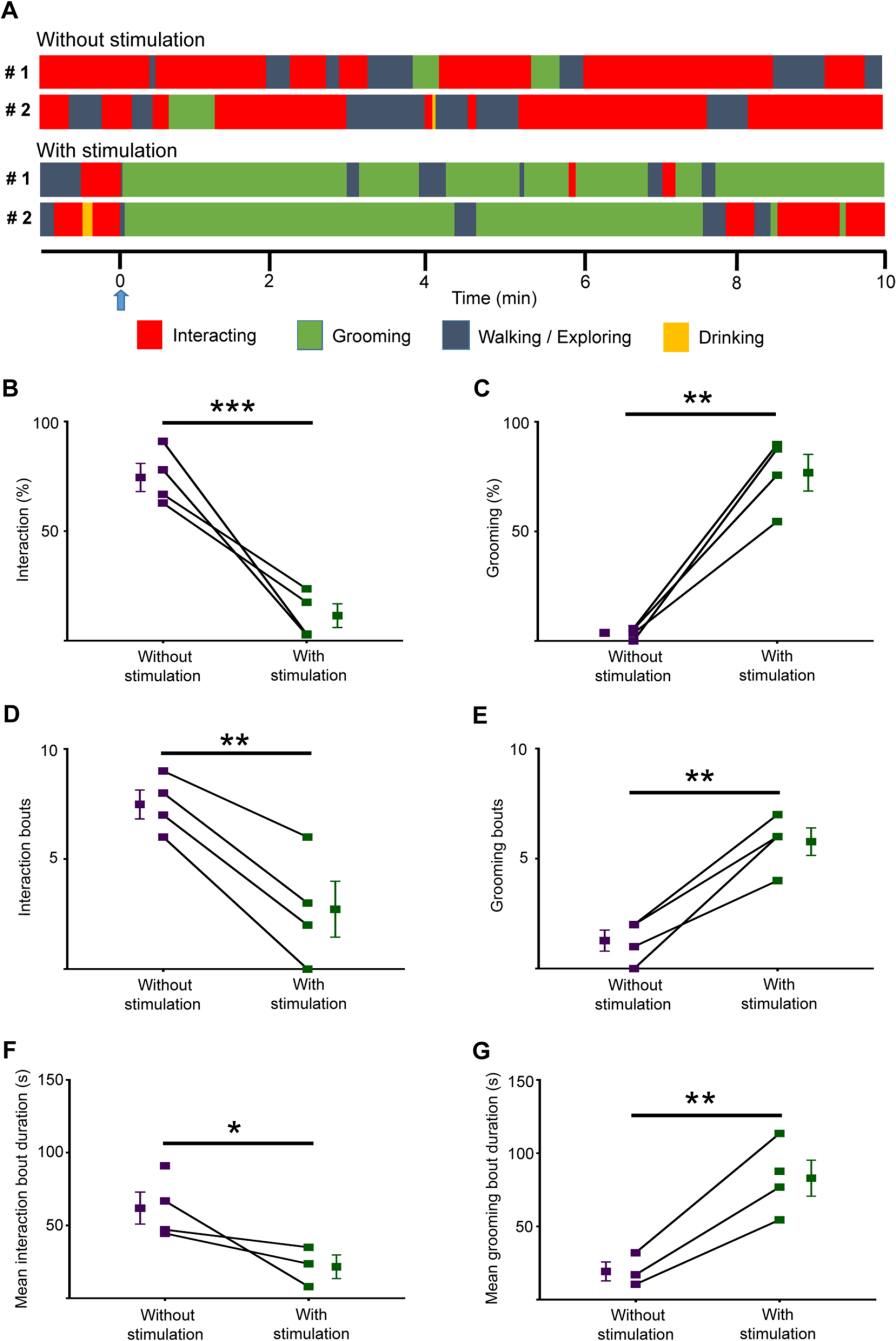
Optogenetic activation of PVH^AVP^ neurons causes self-grooming over social interaction with female mice. **A** Time courses of behaviors of 2 male mice expressing SSFO-EYFP in PVH^AVP^ neurons after introducing a female mouse without or with blue-light illumination (2 s). The stimulation point is shown by a blue arrow and denoted as 0 min in the time course. **B**–**G** Time spent in social interaction (**B**) or self-grooming (**C**), number of social interaction (**D**) or self-grooming bouts (**E**), Mean duration of social interaction (**F**) or self-grooming bouts (**G**) for 10 min of male–female interaction test. Values are mean ± SEM; n = 4; *p < 0.05, **p < 0.005, ***p < 0.001 by Welch's t-test

We also tested whether PVH^AVP^ neuron-induced self-grooming is dominant over social interaction between male and female mice. When exposed to a female mouse, male mice expressing SSFO-EYFP in PVH^AVP^ neurons chased and tried to interact and groom female mice (Fig. 6A, Additional file 6: Video S6). However, optogenetic activation of PVH^AVP^ neurons significantly prevented male mice from interacting with female mice and instead induced self-grooming (latency: 3.7 ± 0.5 s) (Fig. 6A–G, Additional file 7: Video S7). Thus, this result suggested that activation of PVH^AVP^ neurons overrode the need for social interaction and social-grooming to induce self-grooming.

## Discussion

In this study, we found that activation of PVH^AVP^ neurons immediately induces self-grooming. In addition, inhibition of these neurons reduced naturally occurring self-grooming. Intriguingly, stimulation of PVH^AVP^ neurons forced mice to self-groom instead of engaging in the appropriate adaptive behaviors, such as feeding when hungry or social interaction with female mice.

Under normal physiological conditions, mice spent a significant portion of their waking hours self-grooming. Mice use their tongues to lick their bodies and hairs to keep them clean, scratch their bodies with their paws to relieve itchiness, and nibble on their hairs and bodies to remove dust, foreign materials and parasite [1]. In this way, self-grooming maintains hygiene and reduces the risk of contracting infectious diseases. Furthermore, self-grooming behavior occurs frequently when mice are subjected to emotional stresses such as restraint stress, water spray, exposure to light, and forced swimming [5, 11, 34, 37]. For mice, self-grooming seems a means of relieving emotional stress [1, 4, 5].

Multiple neural circuits in the limbic system and hypothalamus have been reported to regulate self-grooming [5, 11–14]. Mangieri et al. demonstrated that optogenetic activation of Sim1-positive PVH neurons induced self-grooming and competed with hunger-induced feeding [11]. They further reported that glutamatergic PVH → ventral lateral septum (LSv) projections of those PVH neurons lacking CRH, oxytocin, and AVP constitute the major component of this behavioral circuit [11, 12]. On the other hand, PVH^CRH^ neurons have also been demonstrated to promote grooming behavior significantly in mice when optogenetically stimulated [13]. Because some PVH^CRH^ neurons were reported to express AVP, the formal possibility remains that PVH^AVP^ neurons we stimulated in the current study overlapped with PVH^CRH^ neurons, and we observed the same phenomena as those by Füzesi et al. [13]. However, we consider this possibility very unlikely. First, AVP expression in PVH^CRH^ neurons is negligible at the basal conditions and increases after adrenalectomy [38]. In addition, CRH neuron-specific Cre driver mice used in Füzesi et al. demonstrated marginal colocalization (~ 5%) of Cre and AVP expression in the PVH [39]. Furthermore, we confirmed little overlap (~ 4%) between CRH immunoreactivity and PVH^AVP^ neurons we studied.

During PVH^CRH^ stimulation, mice spent ~ 30% of their time grooming [13], much less than the activation of PVH^AVP^ neurons shown in this study. PVH^CRH^ neurons are likely to orchestrate complex behaviors after stress, one of which is self-grooming [13]. On the other hand, the rapid and stereotypical induction of grooming suggested that PVH^AVP^ neurons may be more specialized in triggering self-grooming behavior. PVH^AVP^ neurons enhance the stress-induced ACTH secretion from the anterior pituitary [40]. Therefore, these neurons may be well-positioned to regulate multiple aspects of the stress response. Nevertheless, the rapid induction of self-grooming by PVH^AVP^ activation indicates that this induction was caused by a neural mechanism and was not secondary to the endocrine mechanism. The target brain regions of PVH^AVP^ neurons and the interaction between PVH^CRH^ and PVH^AVP^ neurons in the regulation of self-grooming should be elucidated in future studies.

The increase of paw licking compared to spontaneous self-grooming may implicate the similarity of PVH^AVP^ neuron-induced self-grooming to grooming induced by emotional stress. The patterns of self-grooming are variable and may reflect differences in the context [1, 5, 34]. Mu et al. showed in rats that the patterns are different between self-grooming associated more with physical stress and that with emotional stress, such as body restraint and bright light exposure [5]. Intriguingly, self-grooming induced by restraint and light exposure contained paw licking more than physical stress-induced and spontaneous self-grooming. They also demonstrated that the hippocampal ventral subiculum (VS) → LSv → lateral hypothalamus tuberal nucleus is the circuitry critical for emotional stress-induced grooming. Reportedly, PVH^AVP^ neurons receive direct inputs from the LSv [41]. Moreover, bed nucleus of stria terminalis (BNST) is another stress-responsive region that projects PVH^AVP^ neurons [41] and has been implicated in the regulation of self-grooming [42]. Therefore, these projections to PVH^AVP^ neurons from the LSv and BNST may be involved in stress-induced grooming.

The repetitive self-grooming behavior at the expense of social interaction observed in PVH^AVP^-activated mice was similar to the symptoms of autism spectrum disorder, namely limited social interaction, reduced communication, and repetitive behaviors [43, 44]. Repetitive behaviors are also shared by people with obsessive–compulsive disorder (OCD), who have uncontrollable obsessive thoughts and compulsive behaviors [45, 46]. Repetitive self-grooming and increased paw licking induced by the activation of PVH^AVP^ neurons appears to be comparable to frequent hand-washing in OCD patients. Our findings may also be relevant to the obsessive compulsion shown by people with eating disorders [7, 47, 48]. For instance, anorexia nervosa patients skip food even though they are hungry and show compulsive behaviors [47, 49]. Similarly, PVH^AVP^-induced self-grooming overrode fasting-induced feeding. A previous report that activation of PVH^AVP^ neurons reduces food intake in fasted mice may be better interpreted in the same context [24].

In conclusion, PVH^AVP^ neurons play an essential role in the regulation of self-grooming. Their activation triggers grooming at the expense of other adaptive behaviors such as feeding and social interaction. Thus, our study proposes novel functions of PVH^AVP^ neurons in the maintenance behaviors, stress responses, and the pathophysiology of diseases related to repetitive behaviors. Artificial manipulations of self-grooming levels via PVH^AVP^ neurons and identification of input and output pathways of PVH^AVP^ neurons might promote a better understanding of the physiological meanings of such an intriguing behavior.

## Methods

### Animals

Hemizygous *Avp-Cre* mice bred on the C57BL/6 J background, reported previously [33], were used in the present study. We used 12 to 32-week-old male mice, weighing 26–40 g at the time of surgery. Mice were housed under a 12-h light/12-h dark cycle in a temperature- and humidity-controlled room and provided free access to food and water. All experimental procedures were approved by the appropriate institutional animal care and use committees of Kanazawa University. We made every effort to minimize the number of animals used for the experiments and reduce any pain or discomfort experienced by the mice.

### Generation of recombinant viral vectors

The plasmid *pAAV-EF1a-DIO-SSFO-EYFP* was obtained from Dr. Karl Deisseroth as a gift. The plasmids *pAAV-EF1a-DIO-hM3Dq-mCherry*, and *pAAV-EF1a-DIO-hM4Di-mCherry* were obtained from Dr. Bryan Roth as gifts. The plasmid *pAAV-CAG-FLEX-EGFP* was constructed from plasmid *pGP-AAV-CAG-FLEX-jGCaMP7s-WPRE* (Addgene plasmid #104495, a gift from Dr. Douglas Kim & GENIE Project) by replacing *jGCaMP7s* with *EGFP*.

Using a triple transfection helper-free method, recombinant AAV vectors (AAV2-rh10) were produced and purified as described previously [33]. The titers of recombinant AAV vectors were determined by quantitative real-time PCR (genome copies per mL): AAV*-EF1a-DIO-SSFO-EYFP*, 1.8 × 10^12^; AAV*-CAG-FLEX-EGFP*, 1.0 × 10^13^; AAV*-EF1a-DIO-hM3Dq-mCherry*, 1.2 × 10^13^; AAV*-EF1a-DIO-hM4Di-mCherry*, 1.9 × 10^12^.

### Stereotaxic surgery

Stereotaxic injection of AAV vectors was performed as described previously [50]. Mice were anesthetized first with the mixture of medetomidine hydrochloride (0.3 mg/kg, Zenoaq), midazolam (4 mg/kg, Astellas), and butorphanol tartrate (5 mg/kg, Meiji Seika Pharma). When mice lost consciousness, they were placed in the stereotaxic apparatus, and holes were made in the head skull according to requirements. Using a Hamilton Neuros Syringe, 1 μL of AAV vectors was injected unilaterally or bilaterally in the PVH (0.9 mm posterior, ± 0.3 mm lateral, 4.8 mm ventral, relative to the bregma) at a rate of 0.1 μL/min. After 10 min of rest, the needles were removed from the injection site. For optogenetic experiments, an optic fiber (200 μm core, N.A. 0.39, 6 mm, ferrule 1.25 mm, FT200EMT-CANNULA; Thorlabs) was implanted above the PVH (0.9 mm posterior, 0.3 mm lateral, 4.5 mm ventral, relative to the bregma), and then secured to the skull and skin with the dental cement. After the surgical procedure, mice were administered with atipamezole hydrochloride (0.3 mg/kg, Zenoaq) to regain consciousness. Mice were housed individually after surgery and allowed to recover for at least two weeks before starting the experiments.

### Slice electrophysiology

Slice electrophysiology was performed as described previously [51]. We expressed SSFO-EYFP, hM3Dq-mCherry, or hM4Di-mCherry in the PVH of *Avp-Cre* mice by focally injecting the corresponding AAV vectors. After 2–4 weeks, the mice were decapitated under deep anesthesia with isoflurane. Brains were extracted and cooled in ice-cold cutting solution containing following compounds in mM concentration: 87 NaCl, 75 sucrose, 25 NaHCO_3_, 1.25 NaH_2_PO_4_, 2.5 KCl, 0.5 CaCl_2_, 7 MgCl_2_, and 10 D( +)-glucose, bubbled with O_2_ 95% and CO_2_ 5%. Coronal brain slices of 250 μm thickness containing PVH were prepared with a vibratome (NLS-MT, Dosaka EM). The brain slices were incubated at room temperature for at least 1 h in artificial cerebrospinal fluid (ACSF) containing the following compounds in mM concentration: 125 NaCl, 26 NaHCO_3_, 1.25 NaH_2_PO_4_, 2.5 KCl, 2 CaCl_2_, 1 MgSO_4_, and 10 D( +)-glucose, bubbled with O_2_ 95% and CO_2_ 5%. Then the slices were transferred to a recording chamber on a fluorescent microscope stage and continuously perfused with ACSF. EYFP, hM3Dq-mCherry, or hM4Di-mCherry-expressing neurons were identified in the PVH for recording. Cell-attached and whole-cell patch-clamp recordings were performed at 31 °C with borosilicate glass electrodes (4–6 MΩ) prepared by a micropipette puller (P-97, Sutter Instrument) and filled with an internal solution containing the following (mM): 125 K-gluconate, 10 HEPES, 0.2 EGTA, 4 NaCl, 2 MgCl_2_, 4 ATP, 0.4 GTP, and 10 phosphocreatine, pH 7.3, adjusted with KOH. A combination of an amplifier (EPC 10/2, HEKA) and Patch master software (HEKA) was used to control membrane voltage, data acquisition, and triggering of light pulses. To activate SSFO-EYFP, blue (470 nm) light was generated from a solid-state light illuminator (Spectra X light engine, Lumencor). For in vitro hM3Dq-mcherry activation and hM4Di-mcherry inhibition purpose, 10 µM CNO was bath applied during slice electrophysiology.

### Immunostaining

Intracardial perfusion, preparation of serial brain sections, and double immunostaining were performed as described previously [33]. Mice were anesthetized and then fixed by intracardiac perfusion with 4% paraformaldehyde (PFA) in PBS. Serial coronal brain sections of 30 μm thickness were prepared with a cryostat (CM1860, Leica) and collected in 4 series—one of which was further immunostained. Primary antibodies used were guinea pig anti-AVP antibody (1:5000; T-5048, Peninsula Laboratories), rabbit anti-CRH antibody (1:2000; HAC-HM04-01RBP90, the joint/usage research program of the Institute for Molecular and Cellular Regulation, Gunma University for anti-CRH antibody), rabbit anti-GFP antibody (1:1000; A11122, Thermo Fisher Scientific), rat anti-GFP antibody (1:800; 04404-84, Nacalai Tesque), and rabbit anti-c-Fos antibody (1:5000; ABE457, Merck Millipore). Secondary antibodies used were Alexa Fluor 488-conjugated anti-rabbit IgG antibody (1:2000; A-21206, Thermo Fisher Scientific), Alexa Fluor 488-conjugated anti-rat antibody (1:800; A-21208, Thermo Fisher Scientific), Alexa Fluor 594-conjugated anti-guinea pig IgG antibody (1:2000; 11076, Thermo Fisher Scientific), and Alexa Fluor 594-conjugated anti-rabbit IgG antibody (1:2000; A-21207, Thermo Fisher Scientific). Images were taken by an epifluorescence microscope (BZ-9000, Keyence) or a confocal microscope (Fluoview Fv10i, Olympus).

### Optogenetics

For optogenetic studies, the viral vector AAV*-EF1a-DIO-SSFO-EYFP* was focally injected in the PVH of *Avp-Cre* mice, and optic fiber was placed above the PVH, as described above. After at least two weeks from the surgery, individual mice were transferred to the acrylic cage and habituated for at least three days before starting the optogenetic experiment. For blue-light delivery, the implanted optic fiber was connected to an optical cable at least one day before optogenetic stimulation. Then, mice were subjected to a light pulse (2 s, 473 nm, 1–2 mW/mm^2^ at the tip of the optic cable; DL-473 laser, Rapp OptoElectronic) and videorecorded their behavior. Optogenetic experiments were conducted between ZT4 and ZT11. To confirm that PVH^AVP^ neurons were activated by blue-light illumination, we examined cFos expression in these neurons. To do this, two 2-s blue-light pulses were delivered with an interval of 20 min, and then the mice were perfused 70 min after the second light pulse.

To analyze the patterns of self-grooming behavior, four different phases of grooming activities were defined, including paw licking, face/head grooming, body grooming, and leg/tail/genital grooming, according to conventional protocol [1, 5, 34]. To obtain data for spontaneous self-grooming, we videorecorded behavior of *Avp-Cre* mice for 2–3 h between ZT3 and ZT11. The time spent in each phase was expressed as a percentage of the total grooming time, because the grooming time varied from mouse to mouse.

### Chemogenetics

For chemogenetic activation experiment, mCherry-tagged hM3Dq, an excitatory DREADD, was expressed in PVH^AVP^ neurons unilaterally by focally injecting the AAV vector AAV*-EF1a-DIO-hM3Dq-mCherry* in the PVH of *Avp-Cre* mice. For chemogenetic inhibition experiment, the AAV vector AAV*-EF1a-DIO-hM4Di-mCherry* was injected in the PVH bilaterally. Mice were administered with CNO (5 mg/kg body weight; 34233-69-7, Cayman Chemical) or saline intraperitoneally (i.p.) 20 min before starting the videorecording. Mice were videorecorded from ZT3 to ZT4 for the chemogenetic activation study, or ZT11 to ZT12 for the chemogenetic inhibition study. Each mouse received one saline and one CNO administrations in an alternating manner at an interval of 3 d. Mice were habituated to i.p injection for at least three consecutive days before starting the experiment.

### Food deprivation test

After habituation in the acrylic cage for at least three days, individual mice with an optic fiber implant and SSFO expression in PVH^AVP^ neurons were food-deprived for approximately 24 h. Then mice were videorecorded for 10 min during refeeding. After at least 7 d with free access to food, mice were given the same food deprivation-refeeding protocol except for a 2-s blue-light illumination to PVH^AVP^ neurons at the onset of refeeding. Experiments were conducted between ZT4 and ZT11.

### Male–female interaction test

Initially, individual male mice with an optic fiber implant and SSFO expression in PVH^AVP^ neurons were habituated in the acrylic cage for at least 3 days. Then, a female wildtype mouse was introduced in the cage, and the behavior of mice were videorecorded for 10 min. After being housed singly for at least 1–2 days, mice were given the same male–female interaction protocol except for a 2-s blue-light illumination to PVH^AVP^ neurons at the onset of the introduction of female mice. Experiments were conducted between ZT4 and ZT11.

### Statistical analysis

As described in the respective figure legend, statistical analyses included Welch's t-test and paired t-test, performed using Prism 7.0 software (GraphPad). All data are presented as mean ± SEM. p < 0.05 was considered statistically significant.

## Supplementary Information

**Additional file 1: Figure S1.** Stimulation of SSFO in PVH^AVP^ neurons increases their firing frequency in slices. **Figure S2.** Patterns of spontaneous and optogenetically-induced self-grooming. **Figure S3.** Targeted PVH^AVP^ neurons constitute a population distinct from PVH^CRH^ neurons. **Figure S4.** Stimulation of hM3Dq or hM4Di in PVH^AVP^ neurons increases or decreases their firing frequency in slices.

**Additional file 2.Video S2.** Optogenetic activation of SSFO-EYFP-expressing PVH^AVP^ neurons induces self-grooming in a freely moving mouse.

**Additional file 3.Video S3.** Blue-light illumination to EGFP-expressing PVH^AVP^ neurons does not induce self-grooming in a freely moving mouse.

**Additional file 4.Video S4.** A mouse shows voracious feeding after fasting.

**Additional file 5. Video S5.** Optogenetic activation of SSFO-EYFP-expressing PVH^AVP^ neurons causes self-grooming over voracious feeding induced by fasting.

**Additional file 6. Video S6.** Social interaction of a male mouse with a female mouse.

**Additional file 7. Video S7.** Optogenetic activation of PVH^AVP^ neurons induces self-grooming over social interaction with a female mouse.

## Data Availability

All relevant data are within the paper and supporting information files.

## Abbreviations

PVH: Paraventricular hypothalamic nucleus
AVP: Arginine vasopressin
CRH: Corticotropin-releasing hormone
ACTH: Adrenocorticotropic hormone
ICV: Intracerebroventricular administration
SSFO: Stable step-function opsin
EYFP: Enhanced yellow fluorescent protein
EGFP: Enhanced green fluorescent protein
DREADD: Designer receptors exclusively activated by designer drugs
hM3Dq: Human M3 muscarinic cholinergic Gq-coupled receptor
hM4Di: Human M4 muscarinic cholinergic Gi-coupled receptor
OCD: Obsessive–compulsive disorder
