# Supplementary material for: Paraventricular hypothalamic vasopressin neurons induce self-grooming in mice
Source: Mol Brain. 2022 May 23;15:47. doi: 10.1186/s13041-022-00932-9 (PMC9125887; doi:10.1186/s13041-022-00932-9)
Supplement: Supplementary file 1 — Additional file 1: Figure S1. Stimulation of SSFO in PVHAVP neurons increases their firing frequency in slices. Figure S2. Patterns of spontaneous and optogenetically-induced self-grooming. Figure S3. Targeted PVHAVP neurons constitute a population distinct from PVHCRH neurons. Figure S4. Stimulation of hM3Dq or hM4Di in PVHAVP neurons increases or decreases their firing frequency in slices. [file 13041_2022_932_MOESM1_ESM.pdf]

Paraventricular hypothalamic vasopressin neurons induce self-grooming in mice

Md Tarikul Islam[1], Takashi Maejima[1], Ayako Matsui[1], Michihiro Mieda[1,*]

[1]Department of Integrative Neurophysiology, Graduate School of Medical Sciences, Kanazawa University, 13-1 Takara-machi, Kanazawa, Ishikawa 920-8640, Japan.

[*]Correspondence author: mieda@med.kanazawa-u.ac.jp

This file includes:

Figures S1-S4

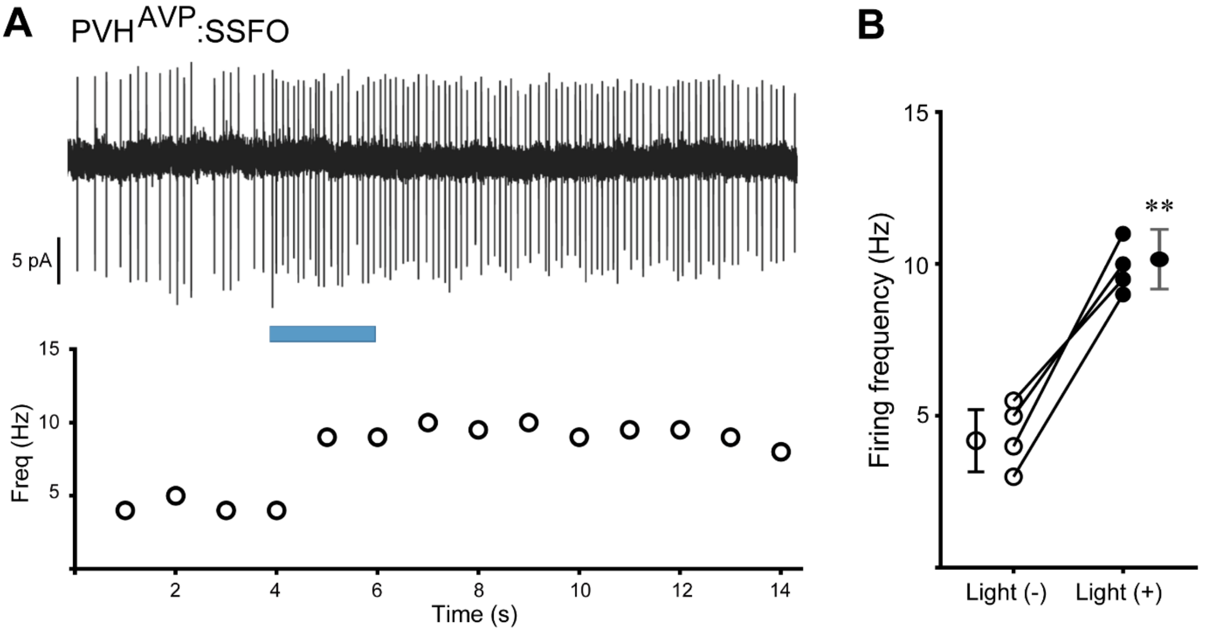

Figure S1. Stimulation of SSFO in PVH^{AVP} neurons increases their firing frequency in slices. (A) Cell-attached recording from an SSFO-EYFP-expressing PVH^{AVP} neuron. Blue light illumination (2 s) increased its firing frequency. The lower graph shows the change in firing frequency. (B) Summary plots showing the blue light effects on the firing frequency of PVH^{AVP} neurons. Welch's t-test; ***p*<0.005.

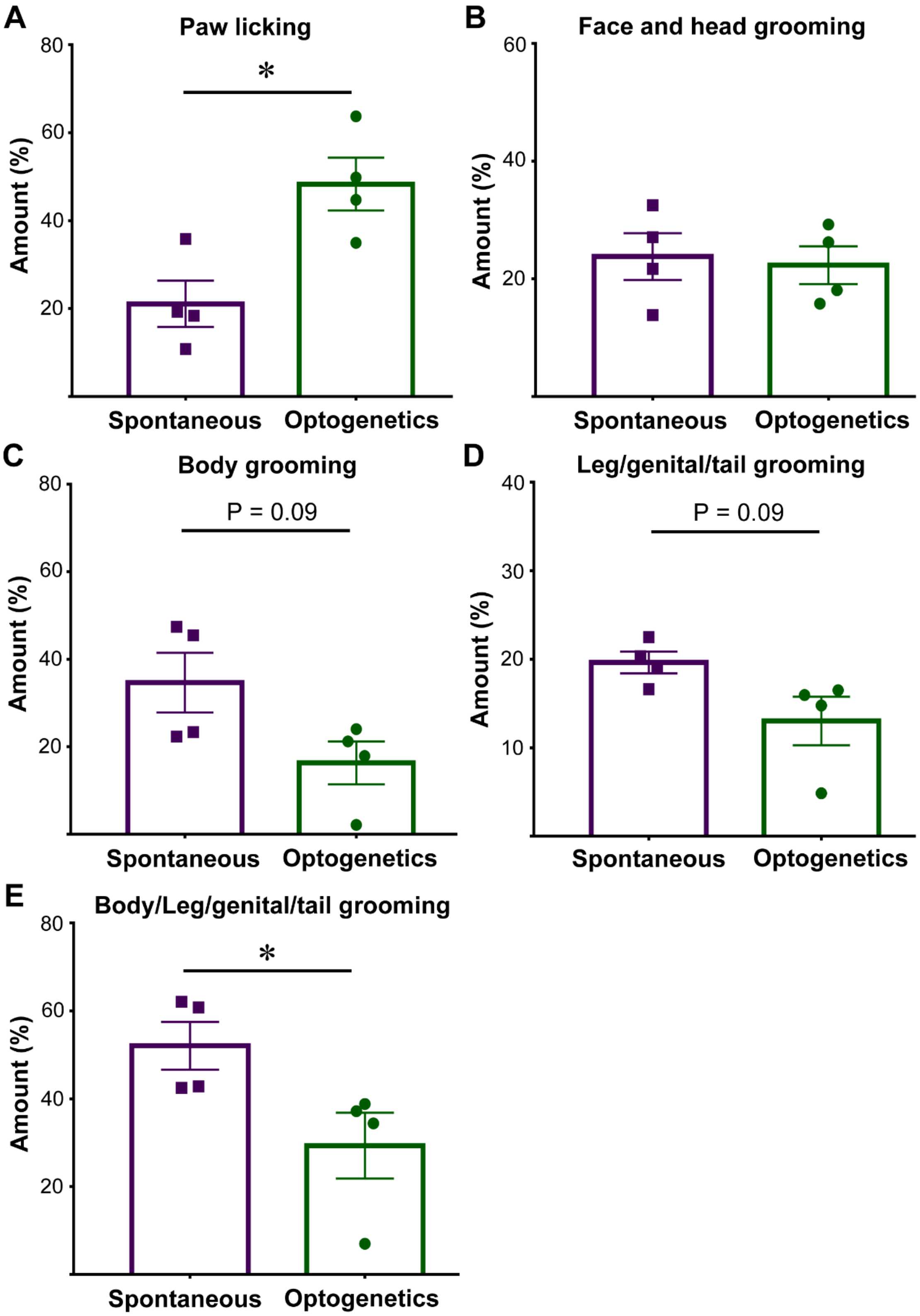

Figure S2. Patterns of spontaneous and optogenetically-induced self-grooming. The proportion of paw licking (A), face/head grooming (B), body grooming (C), leg/genital/tail grooming (D), or body/leg/genital/tail grooming (E, sum of C and D) during spontaneous and PVH^{AVP} neuron-induced self-grooming. Welch's t-test; n = 4; **p*<0.05.

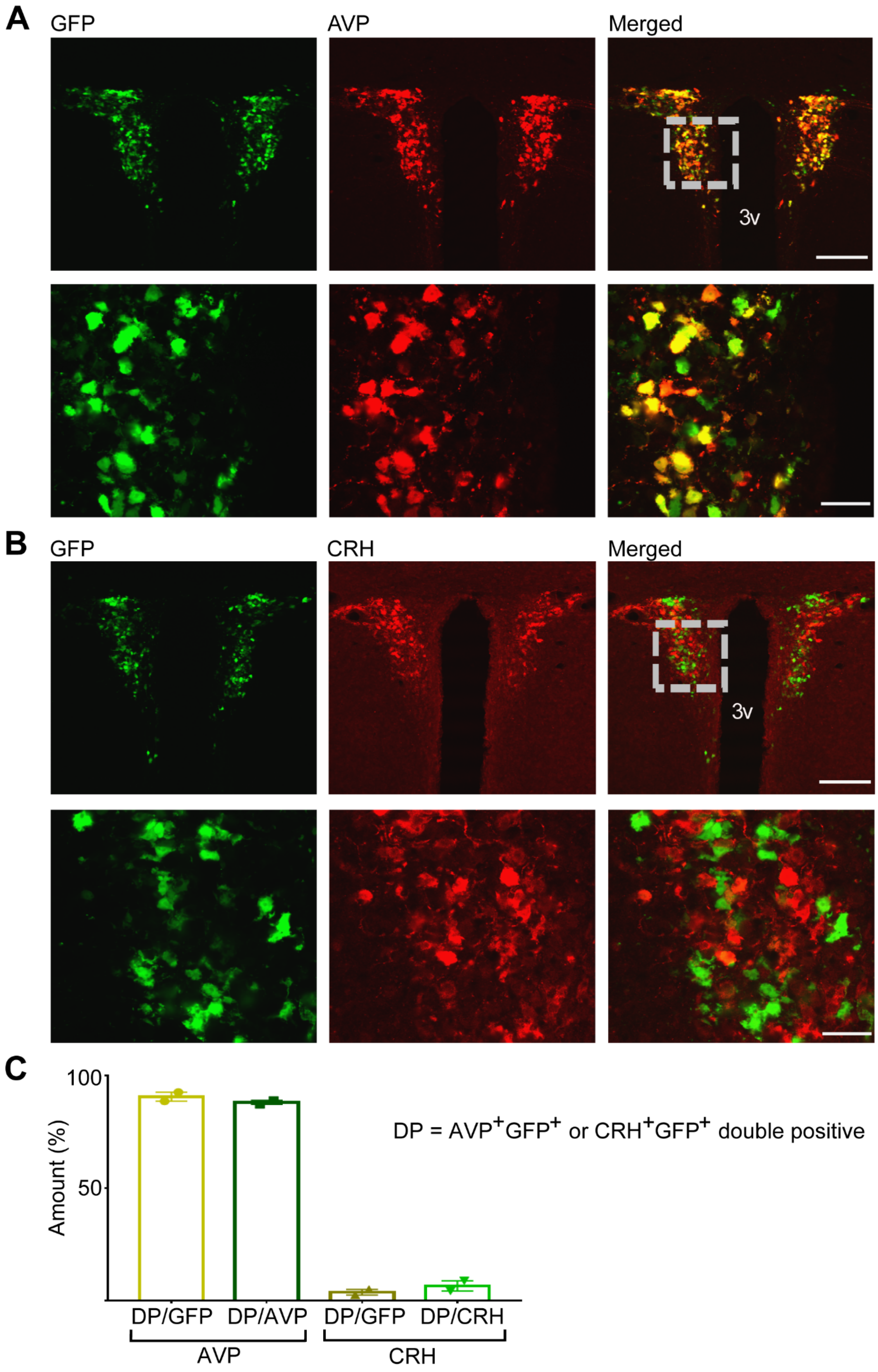

A
GFP
AVP
Merged
3v
B
GFP
CRH
Merged
3v
C
100
50
Amount (%)
DP/GFP
DP/AVP
DP/GFP
DP/CRH
AVP
CRH
DP = AVP+GFP+ or CRH+GFP+ double positive

Figure S3. Targeted PVH^{AVP} neurons constitute a population distinct from PVH^{CRH} neurons. (A, B) Representative coronal brain sections containing the PVH prepared from *Avp-Cre* mice with a focal injection of AAV-*CAG-FLEX-EGFP* in the PVH. Sections were double-stained with anti-AVP (red) and anti-GFP (green) antibodies (A), or anti-CRH (red) and anti-GFP (green) antibodies (B). 3v, third ventricle. (Scale bar: 200 μm for upper panels or 50 μm for lower panels). (C) Proportions of AVP$^+$:EGFP$^+$ cells to EGFP$^+$ cells [DP/EGFP$^+$] or to AVP$^+$ cells [DP/AVP$^+$], and those of CRH$^+$:EGFP$^+$ cells to EGFP$^+$ cells [DP/EGFP$^+$] or to CRH$^+$ cells [DP/CRH$^+$]. DP: AVP$^+$GFP$^+$ or CRH$^+$GFP$^+$ double positive. n = 2.

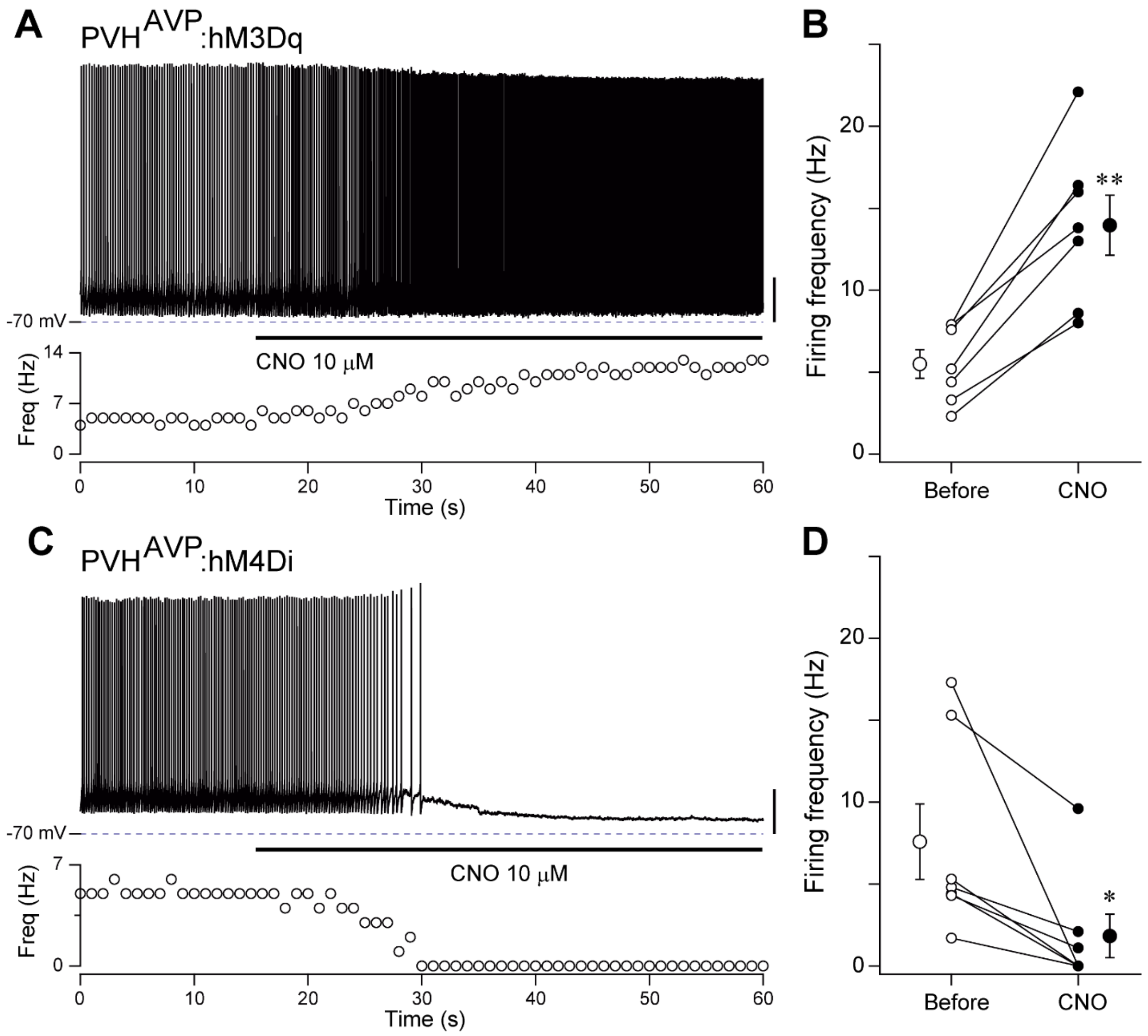

Figure S4. Stimulation of hM3Dq or hM4Di in PVH^{AVP} neurons increases or decreases their firing frequency in slices. (A) Whole-cell current-clamp recording from an hM3Dq-mCherry-expressing PVH^{AVP} neuron. Bath application of 10 μM CNO increased its firing frequency. The lower graph shows the change in firing frequency. (B) Summary plots showing the CNO effects on the firing rate in the hM3Dq-expressing PVH^{AVP} neurons. (C) Representative trace showing that the 10 μM CNO application stopped the firing in an hM4Di-mCherry expressing PVH^{AVP} neuron. (D) Summary plots showing the CNO effects on the firing rate in the hM4Di-expressing PVH^{AVP} neurons. (A, C) Scale bars, 20 mV. (B, D) Summary values are mean ± SEM. Paired t-test; *p<0.05, **p<0.005.
